# The role of NMDA receptors in rat propofol self-administration

**DOI:** 10.1186/s12871-020-01056-0

**Published:** 2020-06-15

**Authors:** Bei-ping Chen, Xi-xi Huang, Dong-mei Dong, Hui Wu, Tian-qi Zhu, Ben-fu Wang

**Affiliations:** 1 Department of Anesthesiology, Second Affiliated Hospital and Institute of Neuroendocrinology, Wenzhou Medical University, 109 Xueyuan Western Road, Wenzhou City, 325000 Zhejiang Province China; 2 Department of Anesthesiology, First Affiliated Hospital of Wenzhou Medical University, Shangcai village, Nanbaixiang town, Ouhai District, Wenzhou City, 325000 Zhejiang Province China

**Keywords:** Propofol, Reinforcement, NMDA receptor, Self-administration, MK-801

## Abstract

**Background:**

Propofol is among the most frequently used anesthetic agents, and it has the potential for abuse. The N-methyl-D-aspartate (NMDA) receptors are key mediators neural plasticity, neuronal development, addiction, and neurodegeneration. In the present study, we explored the role of these receptors in the context of rat propofol self-administration**.**

**Methods:**

Sprague-Dawley Rats were trained to self-administer propofol (1.7 mg/kg/infusion) using a fixed-ratio (FR) schedule over the course of 14 sessions (3 h/day). After training, rats were intraperitoneally administered the non-competitive NDMA receptor antagonist MK-801, followed 10 min later by a propofol self-administration session.

**Results:**

After training, rats successfully underwent acquisition of propofol self-administration, as evidenced by a significant and stable rise in the number of active nose-pokes resulting in propofol administration relative to the number of control inactive nose-pokes (*P* < 0.01). As compared to control rats, rats that had been injected with 0.2 mg/kg MK-801 exhibited a significantly greater number of propofol infusions (F (3, 28) = 4.372, *P* < 0.01), whereas infusions were comparable in the groups administered 0.1 mg/kg and 0.4 mg/kg of this compound. In addition, MK-801 failed to alter the numbers of active (F (3, 28) = 1.353, *P* > 0.05) or inactive (F (3, 28) = 0.047, P > 0.05) responses in these study groups. Animals administered 0.4 mg/kg MK-801 exhibited significantly fewer infusions than animals administered 0.2 mg/kg MK-801 (*P* = 0.006, *P* < 0.01). In contrast, however, animals in the 0.4 mg/kg MK-801 group displayed a significant reduction in the number of active nose-poke responses (F (3, 20) = 20.8673, *P* < 0.01) and the number of sucrose pellets (F (3, 20) = 23.77, *P* < 0.01), while their locomotor activity was increased (F (3, 20) = 22.812, P < 0.01).

**Conclusion:**

These findings indicate that NMDA receptors may play a role in regulating rat self-administration of propofol.

## Background

Propofol (2,6-di-isopropylphenol) is an anesthetic agent that has the potential for recreational abuse, as its intake is associated with pleasant and euphoric feelings [1]. Indeed, one anesthesiologist that had been self-administering propofol reported eventual propofol-dependence [2]. Both conditioned place preference and self-administration studies in animals have further confirmed that propofol can provide reinforcement [3–5]. We have previously demonstrated a role for GABA receptors in the ventral tegmental area (VTA) as regulators of propofol self-administration behavior [6]. We further found that the D1R antagonist SCH23390 was able to mediate a simultaneous dose-dependent reduction in propofol self-administration while also decreasing D1R-associated p-ERK/ERK levels in the nucleus accumbens (NAc) [7, 8]. These previous results clearly indicate that propofol is susceptible to abuse, although the mechanistic basis for this susceptibility remains uncertain.

Glutamate directly and indirectly controls drug addiction via the regulation of the dopaminergic system [9–11]. Glutamatergic inputs within the VTA increase dopamine release within the NAc and result in increased dopaminergic cell activity [12]. Glutamate can further facilitate dopaminergic transmission, likely via acting at the presynaptic stage to regulate dopamine release [13]. Alcohol dependence and withdrawal have both been linked to elevated levels of glutamate and associated neuroadaptation in rodent models and in humans [14]. Dependence on other agents such as heroin, cocaine, and nicotine is similarly associated with clear changes in extracellular levels of glutamate in the VTA, NAc, PFC, and striatum [15, 16]. Propofol has also been suggested to alter glutamate levels, with multiple studies having demonstrated that propofol can inhibit glutamate receptor-mediated excitatory synaptic transmission [17, 18].

N-methyl-D-aspartate (NMDA) receptors are glutamate receptors that control learning, neuroplasticity, and memory, but that also contribute to drug addiction. Studies of chronic neuronal exposure to ethanol have demonstrated that NMDA receptor activity can be directly influenced by ethanol [19], with similar findings having been made in vivo [20]. The non-competitive NMDA antagonist MK-801 can be systemically administered to animals, and such administration has been shown to suppress the development of opioid dependence and opioid tolerance [21]. MK-801 can similarly inhibit the onset of morphine withdrawal syndrome [22], interfere with the acquisition of cocaine self-administration behaviors [23], and alter conditioned place preference study outcomes [24]. NMDA receptor antagonism can also alter responses to other psychostimulant compounds including amphetamines and alcohol [25]. Work in vitro has shown that propofol can inhibit NMDA receptors, thereby causing hallucinations that are known to occur following propofol anesthetization [26]. Additional work suggests that when used at an anesthetic dose, propofol can disrupt NDMA receptor agonist-induced calcium entry into cells [27, 28]. These findings thus strongly suggest that there is a link between the NMDA receptor and propofol dependence.

Based on these prior findings, we hypothesized that NMDA receptors play a role in regulating propofol addiction. To test this possibility, we developed a rat propofol self-administration model system. These model rats were then intraperitoneally administered MK-801, after which we assessed the effects of such administration on both general and propofol-specific activity in these animals.

## Methods

### Subjects

In total, 80 male Sprague-Dawley rats (280–300 g) from the Slac Laboratory Animal Center of Shanghai (Shanghai, China) were obtained and individually housed in a temperature-controlled facility with a 12 h light/dark cycle and free food/water access. All animal protocols were approved by the Animal Care and Use Committee of Wenzhou Medical University (wydw2015–0121, Zhejiang, China). At the end of the study, animals were euthanized using sodium pentobarbital (100 mg/kg, IP).

### Drugs

Animals were injected intravenously with propofol (10 mg/mL; Diprivan, Astrazeneca, Italy), with drug solutions being prepared immediately prior to use. The 1.7 mg/kg per infusion dose of propofol used in this self-administration model was chosen based on a previous report [29]. MK-801 was obtained from Sigma-Aldrich (USA), and was prepared using sterile water.

### Apparatus

Twelve customized plexiglass operant boxes (Ningbo Addiction Research and Treatment Center, China) were used for propofol training studies, as detailed in previous reports [30]. These boxes contained two nose-poke holes at a height of 5 cm, with each hole containing a yellow LED light. In addition, a larger light was present on the wall over these holes (28 V, 0.1 mA). Drugs were delivered to animals via Tygon tubing, with a leash assembly being used to protect the tubing and a plastic swiveling apparatus being used to guide the tubing through the ceiling. Animals wore jackets containing a customized fluid connector that attached to the leash assembly. A 5 mL syringe pump was attached to the tubing, delivering fluids at a 1.2 mL/min rate. A MED Associates interface on an IBM PC was used for experimental control, which was achieved with software written internally using Borland Delphi 6.0.

### Surgery

Sodium pentobarbital (50 mg/ml) was used to anesthetize animals, after which incisions were made on the chest above the right jugular vein and at the mid-scapular level on the back. Next, a chronically indwelling silastic jugular cannula was implanted such that it extended from the back of the animal [30]. A total of 0.2 mL of a saline-heparin solution (25 U/mL heparin) was flushed through these cannulas each day in order to maintain patency, while infections were prevented via the daily administration of sodium penicillin for 5 days after surgery. Prior to study initiation, animals were given a 7 day post-operative recovery period [29].

### Propofol self-administration

Propofol self-administration training was performed as in previous reports [30]. Briefly, after surgical recovery animals were placed into the customized operant boxes and infusion lines were attached as appropriate. At the start of each session, the yellow LED within the active nose-poke hole was lit. Each session started with the illumination of yellow light inside the active nose-poke hole, then they were administered one 1.7 mg/kg infusion of propofol following completion of the ratio requirement (fixed ratio = 1) in the active nose-poke. Fixed ratio = 1(FR1) mean the one touch the active nose-poke hole allowed one injection of propofol. This administration was paired to a 5-s illumination of the house light in addition to the noise of the infusion pump apparatus. After this time, a 15 s timeout period was activated during which responses were recorded but did not alter drug administration. At the end of this period, the yellow LED within the active nose-poke hole was again illuminated. There were no consequences for responses to the inactive nose-poke hole. Sessions were allowed to continue until either 3 h had elapsed or until 100 propofol infusions had been administered.

### Specific experiments

The 32 rats in this study underwent propofol self-administration training once per day (1.7 mg/kg per infusion). Once rats exhibited a stable response (±5%) for 5 consecutive days, they were intraperitoneally administered MK-801 (0, 0.1, 0.2, or 0.4 mg/kg; *n* = 8/group) 10 min before starting the next session, as determined based on previous reports [31]. Animal responses over a 1 h self-administration period were then recorded, after which animals were euthanized.

### Sucrose self-administration

For sucrose administration tests, rats were placed in 30 × 20 × 24 cm operant conditioning chambers and trained according to an FR1 schedule using food reinforcement (45 mg pellets; Noyes, NH, USA) over a period of 7 days. For these chambers, two nose-poke holes were present with a light above each and a food dispenser located between the two holes. In addition, a house light was present on the opposite wall. Food pellets were delivered only in response to the pressing of the active hole during nose-poke tests, while pressing the inactive hole elicited no response. Tests were allowed to proceed either for 3 h or until 100 food pellets had been acquired.

After rats exhibited stable sucrose acquisition responses, they were intraperitoneally injected with MK-801 (0, 0.1, 0.2, or 0.4 mg/kg) 10 min prior to the next session. The numbers of active/inactive pose-poke responses and the number of sucrose pellets obtained were then recorded over the course of a 1 h session period.

### General activity test

In order to explore the ability of MK-801 to non-specifically influence general activity in rats, animals were intraperitoneally administered MK-801, after which locomotion in a novel context was analyzed. In total, 24 naive rats (*n* = 6/group) were administered MK-801 (0, 0.1, 0.2, or 0.4 mg/kg), with their total distance traveled (cm) then being measured and analyzed with the MED Associates SOF-811 Open-field Activity Software.

### Statistical analysis

The number of infusions or responses for active and inactive holes during self-administration testing was analyzed using a one-way analysis of variance (ANOVA) or a two-way (hole, treatment) repeated-measures ANOVA. Newman-Keuls multiple comparison tests with an alpha level of 0.05 or 0.01 were used for post-hoc comparisons of group means.

## Results

### Acquisition of propofol self-administration in rats

**Fig. 1:**
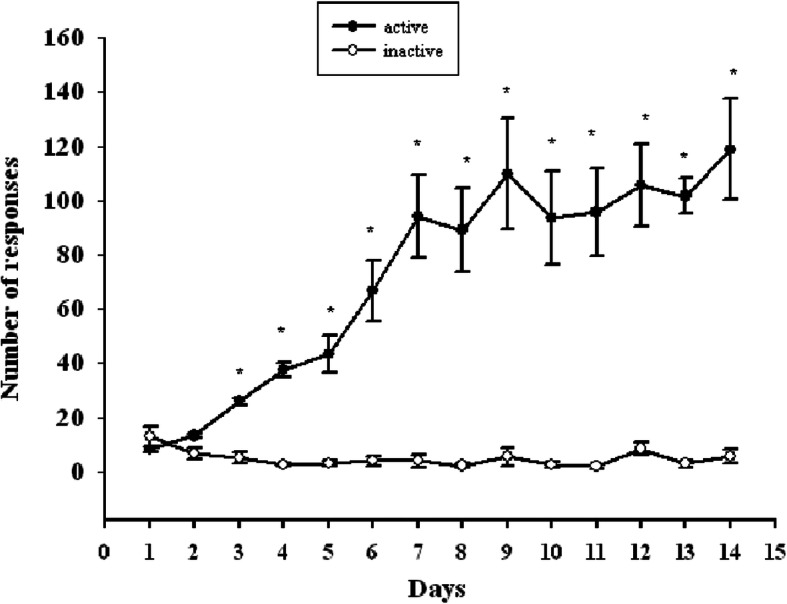
The X-axis represents the number of days of propofol self-administration training. The Y-axis represents the number of responses, with ● representing active nose-pokes and ○ representing inactive nose-poke. As the number of training days increases, the numbers of active nose-pokes rose significantly relative to the number of inactive nose-pokes in study animals (*P* < 0.01), rats exhibited stable responses after a total of 14 training sessions

Over the course of propofol self-administration training, the numbers of active nose-pokes rose significantly relative to the number of inactive nose-pokes in study animals (*P* < 0.01)(Fig. 1). Using an FR1 schedule of propofol self-administration (1.7 mg/kg per infusion), rats exhibited stable responses after a total of 14 training sessions.

### The impact of MK-801 on propofol self-administration

**Fig. 2:**
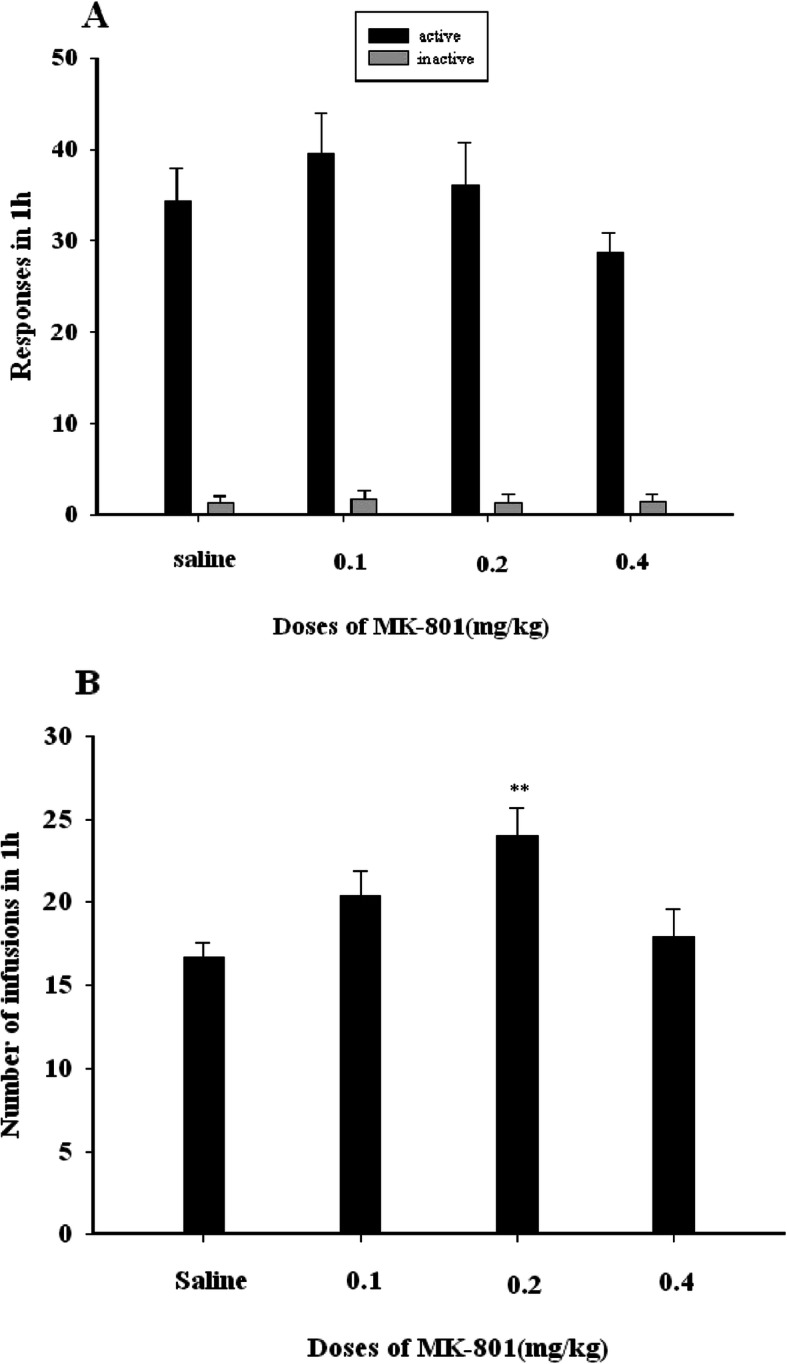
**a** The X-axis represents the dose of MK-801(mg/kg). The Y-axis represents the number of responses in 1 h of propofol self-administration. Black bars represent active nose-pokes, while gray bars represent inactive nose-pokes. Compared with the saline group, MK-801(0.1, 0.2, 0.4 mg/kg) groups have no significant differences in numbers of active nose-poke (F (3, 28) = 1.353, *P* > 0.05) (**a**) or inactive nose-poke (F (3, 28) = 0.047, P > 0.05). **b** The X-axis represents the dose of MK-801 (mg/kg). The Y-axis represents the number of propofol infusions in 1 h, Black bars represent the number of propofol infusions in 1 h. Compared with the saline group, a significant difference in the number of infusions per session following MK-801 administration (F (3, 28) = 4.372, *P* < 0.01), 0.2 mg/kg MK-801 significant increased in the number of propofol infusions (*P* = 0.003,P < 0.01)

We observed no significant differences in numbers of active (F (3, 28) = 1.353, *P* > 0.05) (Fig. 2a) or inactive (F (3, 28) = 0.047, P > 0.05) (Fig. 2a) responses among groups when NMDA receptor activity was antagonized. We did, however, observe a significant difference in the number of infusions per session following MK-801 administration (F (3, 28) = 4.372, *P* < 0.01) (Fig. 2B), with a significant increase in number of infusions for animals pre-treated with 0.2 mg/kg MK-801 relative to saline controls (*P* = 0.003,P < 0.01).

### Acquisition of sucrose self-administration responses in rats

**Fig. 3:**
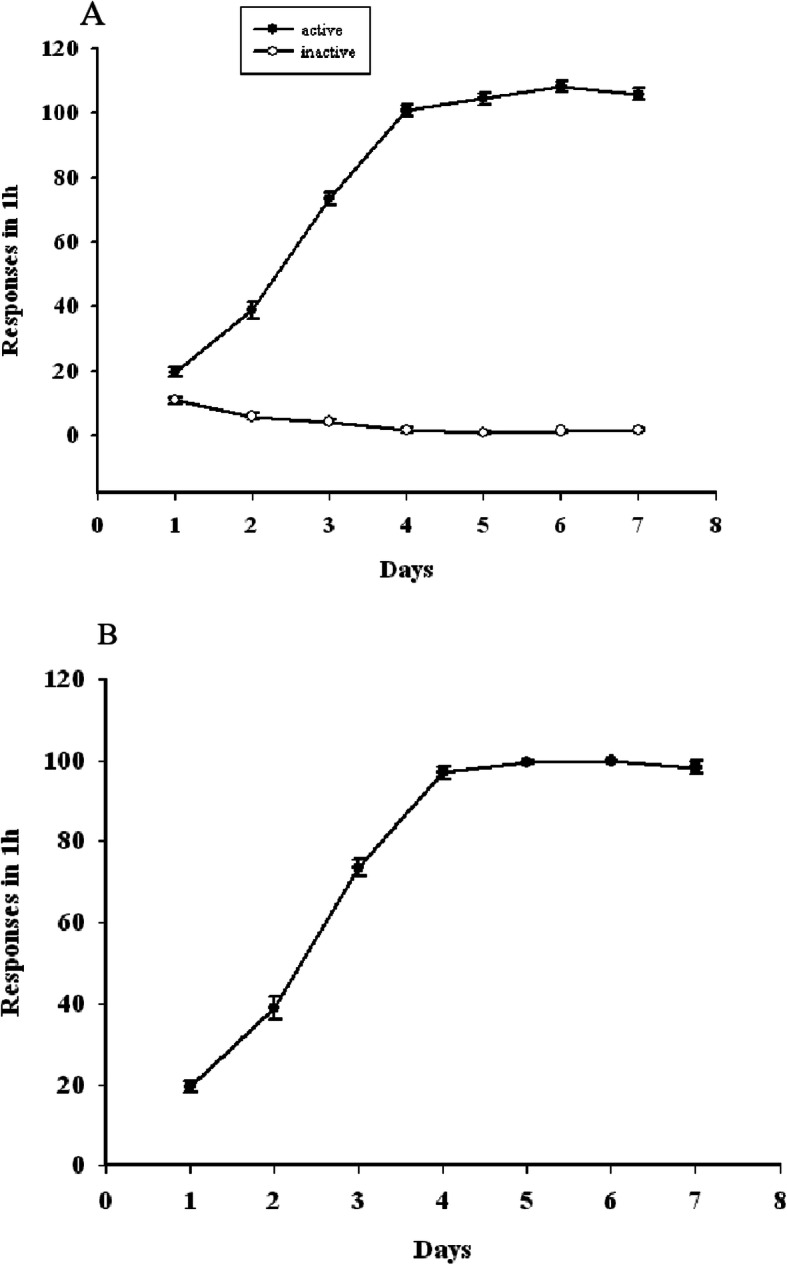
**a** The X-axis represents the number of days of sucrose self-administration training. The Y-axis represents the number of responses, with ● representing active nose-pokes and ○ representing inactive nose-poke. There was a significant increase in the number of active but not inactive nose-poke responses over this training period, rats acquired a stable sucrose self-administration response after 7 total sessions. **b** The X-axis represents the number of days of sucrose self-administration training. The Y-axis represents the number of food pellets acquired in 1 h. After 4 days, rats were stably acquiring 100 food pellets per training session

Using an FR1 schedule, rats acquired a stable sucrose self-administration response after 7 total sessions. There was a significant increase in the number of active but not inactive nose-poke responses over this training period (Fig. 3a). After 4 days, rats were stably acquiring 100 food pellets per training session (Fig. 3b).

### The impact of MK-801 pretreatment on sucrose self-administration

**Fig. 4:**
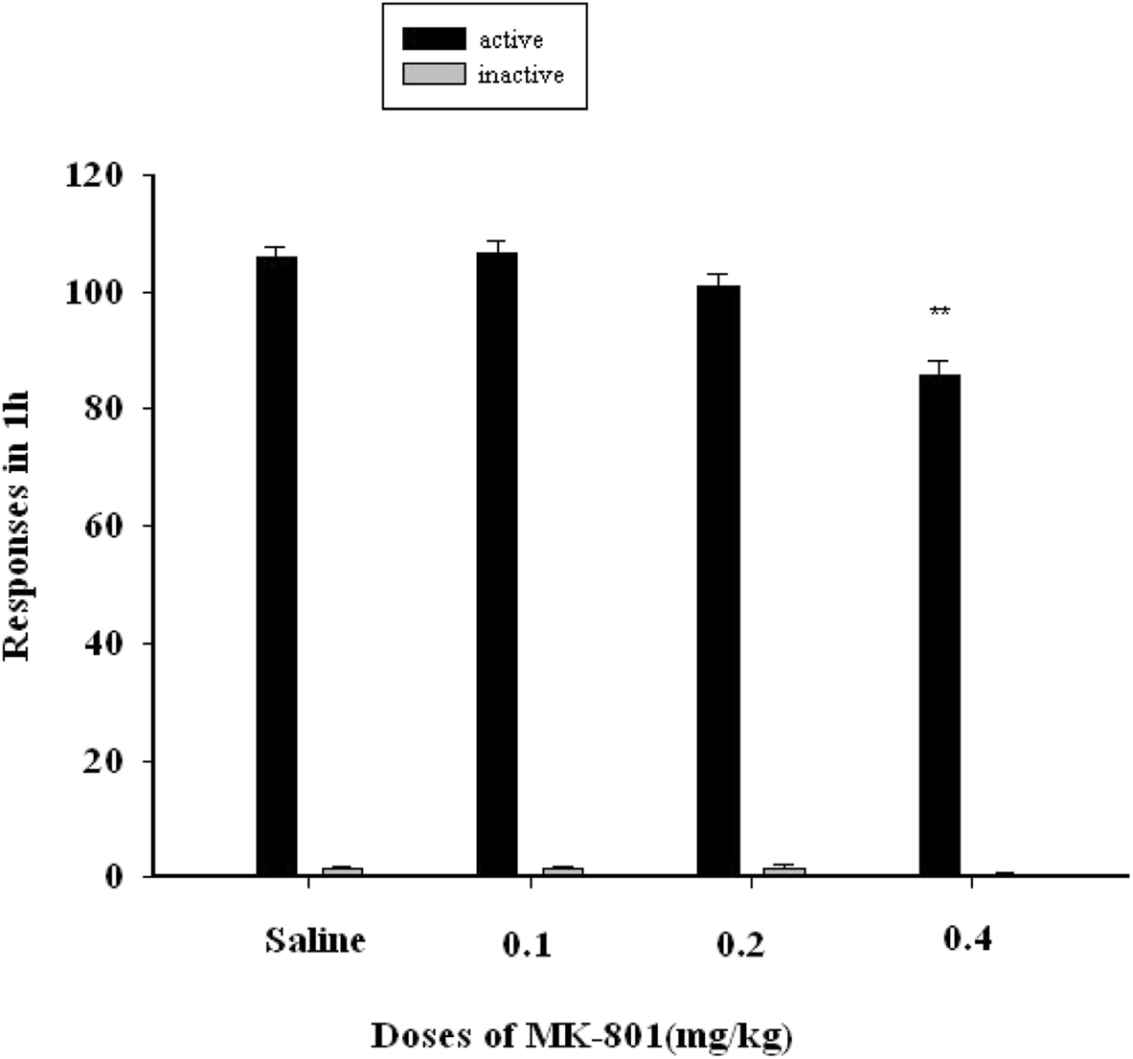
The X-axis represents the dose of MK-801(mg/kg). The Y-axis represents the number of responses in 1 h of sucrose self-administration. Black bars represent active nose-pokes, while gray bars represent inactive nose-pokes. MK-801 (0.1 or 0.2 mg/kg, ip) have no changes in the numbers of active or inactive nose-poke responses in rats sucrose self-administration, 0.4 mg/kg MK-801 dose exhibited a significant reduction in the number of active nose-poke responses (F (3, 20) = 20.8673, P < 0.01)

We observed no changes in the numbers of active or inactive nose-poke responses in rats treated with MK-801 (0.1 or 0.2 mg/kg, ip) during sucrose self-administration testing, whereas animals administered the higher 0.4 mg/kg MK-801 dose exhibited a significant reduction in the number of active nose-poke responses (F (3, 20) = 20.8673, *P* < 0.01), coinciding with a reduction in numbers of sucrose pellets obtained (F (3, 20) = 23.77, P < 0.01)(Fig. 4).

### The impact of MK-801 on rat general activity

**Fig. 5:**
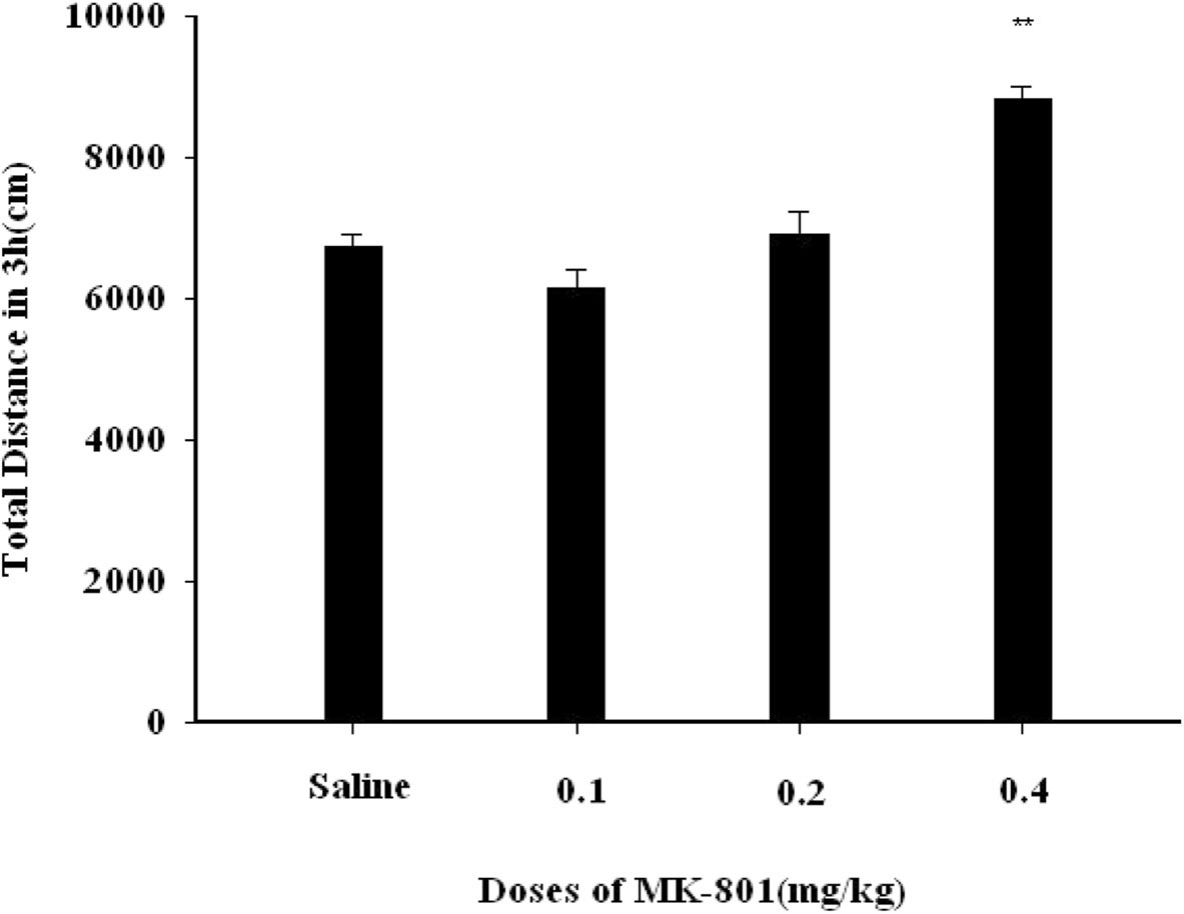
The X-axis represents the dose of MK-801 (mg/kg). The Y-axis represents the total distance covered by rats in 3 h. 0.1 mg/kg or 0.2 mg/kg MK-801 have no changes on general locomotor activity in rats (P > 0.05), whereas the higher 0.4 mg/kg MK-801 dose significantly increased this activity (F (3, 20) = 22.812, P < 0.01

We did not observe any apparent impact of 0.1 mg/kg or 0.2 mg/kg MK-801 on general locomotor activity in rats (*P* > 0.05), whereas the higher 0.4 mg/kg MK-801 dose significantly increased this activity (F (3, 20) = 22.812, P < 0.01)(Fig. 5).

## Discussion

Our results clearly demonstrate that propofol is capable of mediating reinforcement in self-administration studies, consistent with previous studies [6–8]. We found that lower doses of MK-801 (0.1–0.2 mg/kg) were able to increase propofol self-administration infusion numbers in a dose-dependent fashion in rats without impacting general locomotor activity, in line with previous findings. For example, one study has found that MK-801 increases cocaine self-administration breaking points for animals on a progressive-ratio schedule [32]. Recent research suggests that MK-801 disrupts the reconsolidation of cocaine-associated memories during conditioned place preference but not self-administration studies in rats [33]. Another study indicates that MK-801 treatment can increase low rates of operant responding [34]. MK-801 has been shown to facilitate low intracranial self-stimulation (ICSS) rates maintained by low brain-stimulation frequencies in rats [35]. We additionally found that MK-801 at these dose levels did not impact locomotor activity or sucrose-self administration in rats, with our results thus indicating that 0.2 mg/kg MK-801 increases propofol reinforcement.

For the highest MK-801 dose used in this study (0.4 mg/kg), the increase in the number of propofol infusions over control was lower than was observed for the two lower MK-801 doses (Fig. 1b). This is consistent with previous results indicating that at lower doses MK-801 is capable of lowering the reward threshold for brain stimulation, thereby influencing self-administration, whereas higher doses (0.3 mg/kg) are capable of disrupting operant performance [36], consistent with the observed impact on sucrose self-administration in the present study (Fig. 4*P* < 0.01). Higher doses of MK-801 may be inducing continuously increasing effects on brain electric energy, leading to stronger behavioral effects [37], which can lead to increased ataxia in rats [38]. Consistent with this, we observed no impact of lower MK-801 doses on rat general locomotion, whereas the higher 0.4 mg/kg MK-801 dose led to ataxia and increased hovering behavior in our study animals (Fig. 5 P < 0.01).

Multiple mechanisms have the potential to explain the ability of MK-801 to increase propofol rewarding. MK-801 is able to potently bind to the NMDA binding site in a non-competitive manner [39]. As such, MK-801 is able to block NMDA receptor signaling, thereby reducing transduction through glutamatergic nerves. MK-801 has also been shown to enhance the effects of propofol via increasing DA input in the NAc. In microdialysis experiments, the systemic delivery of MK-801 (0.2–0.5 mg/kg) has indeed been shown to result in a marked increase in extracellular DA within the Nac [40]. Another hypothesis proposes that DA signaling may be a dominant mediator of drug addiction, with glutamate signaling playing a less substantial secondary role in this process. Indeed, MK-801 is only able to partially ablate the cocaine-induced expression of c-Fos, whereas the D1 receptor antagonist SCH23390 can completely abolish this induction [41]. Similarly, MK-801 can mediate the partial inhibition of progressive ERK activation in the NAc and dorsal striatum of rats following acute Δ9-tetrahydrocannabinol (THC) administration, while SCH23390 fully blocked this response [42]. We have previously found the D1 dopamine receptors to be linked with ERK activity in the NAc in the context of propofol self-administration [7, 8]. This suggests that NMDAR activity is likely a mediator of propofol self-administration responses, with dopamine signaling being dominant and glutamate signaling playing a secondary role in this process.

In summary, these results suggest that NMDA receptors play a role in regulating propofol self-administration. This study does have certain limitations, such as the lack of inclusion of other NMDAR antagonists or agonists, and a lack of intracerebral microinjection experiments or measurements of ERK protein activation. As such, additional research will be needed to clarify the role of these receptors in propofol addiction.

## Conclusion

These findings indicate that NMDA receptors may play a role in regulating rat self-administration of propofol.

## Data Availability

The datasets used and/or analysed during the current study are available from the corresponding author on reasonable request.

## Abbreviations

NMDA: N-methyl-D-aspartate
FR: fixed ratio
GABA: Gamma-aminobutyric acid
VTA: ventral tegmental area
D1R: Dopamine1 receptor
NAc: nucleus accumbens
PFC: Prefrontal cortex
I.p: Intraperitoneal injection
DA: Dopamine
THC: Δ9-tetrahydrocannabinol
